# Precision management of brain oedema after acute ischaemic stroke

**DOI:** 10.1093/pcmedi/pbac019

**Published:** 2022-07-22

**Authors:** Simiao Wu, Craig S Anderson

**Affiliations:** Department of Neurology, West China Hospital, Sichuan University, Chengdu 610041, China; Centre for Cerebrovascular Diseases, West China Hospital, Sichuan University, Chengdu 610041, China; The George Institute for Global Health, Faculty of Medicine, University of New South Wales, Sydney NSW 2050, Australia; The George Institute for Global Health China, Beijing 100600, China

Dear Editor,

Stroke is a leading cause of death and disability in the world, with acute ischaemic stroke accounting for ∼70% of all cases.^1^ Some degree of brain oedema occurs in all cases of acute ischaemic stroke and, when severe, has major consequences on survival-free of disability. Pathophysiological studies indicate an evolution of cytotoxic oedema, ionic oedema, and vasogenic oedema,^2^ which implies brain oedema is a dynamic process and explains the various terminology used, such as large hemispheric infarction, symptomatic infarct swelling, space-occupying infarction, or malignant middle cerebral artery (MCA) infarction. Heterogeneity in manifestations and sequelae necessitates a new, precision medicine approach to individualizing prevention and treatment of brain oedema after stroke.

Approximately 10% of patients with ischaemic stroke develop symptomatic brain swelling, most occurring within the first few days after symptom onset. Malignant MCA infarction is a fatal stage of brain oedema, with a rapid deterioration in neurological deficits associated with brain oedema, which can evolve into a malignant course with massive brain swelling, transtentorial herniation, and death or disability.^3^ To interrupt such a malignant course, current guidelines recommend that patients aged between 18 and 60 years who develop space-occupying hemispheric infarction within 48 hours, would benefit from decompressive hemicraniectomy (DHC).^4^ Randomized controlled trials have shown that DHC reduces the risk of fatal brain oedema, albeit in selected patients and with some offset price from persistent disability in survivors; however, there is insufficient evidence to inform the appropriate management of patients who present after 48 hours.^4^ More data are needed to determine the benefits and risks of DHC in broader patient groups. Ongoing observational studies will allow greater clarity over clinical and imaging predictors, and of the natural history of malignant brain oedema (e.g. NCT03222024), to inform patient selection and optimal therapeutic windows for the design of interventions studies.

Early identification of patients at a high-risk of developing malignant brain oedema would guide timely interventions and improve outcome prediction. A recent systematic review and meta-analysis included 38 studies involving 3278 patients and analysed 24 clinical factors, seven domains of imaging markers, and 13 serum biomarkers for predicting malignant brain oedema.^5^ Overall, a younger age, more severe neurological deficits, larger infarct volume, and early signs of brain oedema are the reliable early predictors for malignant brain oedema, while successful revascularization is associated with a reduced risk (Table 1). The effect of revascularization on reducing the risk of malignant brain oedema has been further clarified in reperfusion trials. These findings provide mechanistic insights to underpin the benefit of reperfusion treatment, even in patients with large infarction, and imply that proper management of brain oedema may further improve outcomes in patients with very large infarction.

**Table 1.** Factors for prediction and prevention for malignant brain oedema after stroke.

Factors for prediction	Characteristics and treatment effect on malignant brain oedema
**Clinical features**	
Age (years)	22 studies, 2075 patients, pooled MD −4.42, 95% CI −6.63 to −2.22
Admission NIHSS scores	8 studies, 807 patients, range of median score 17 to 20 vs. 5.5 to 15
Depressed consciousness	6 studies, 573 patients, pooled OR 6.65, 95% CI 3.28 to 13.46
**Imaging signs**	
Infarct volume (cm^3^)	13 studies, 929 patients, pooled SMD 2.57, 95% CI 1.82 to 3.31
Hypoattenuation >50% of MCA territory	4 studies, 420 patients, pooled OR 5.33, 95% CI 2.93 to 9.68
Compression of ventricles	5 studies, 526 patients, pooled OR 7.75, 95% CI 2.40 to 25.04
Midline shift	3 studies, 199 patients, pooled OR 13.90, 95% CI 3.13 to 61.82
Compression of basal cisterns	2 studies, 284 patients, pooled OR 19.09, 95% CI 5.00 to 72.93
BBB permeability^9^	1 study, 238 patients, common OR 1.12, 95% CI 1.03 to 1.20, *P* = 0.005
Cerebral blood flow^9^	1 study, 238 patients, common OR 0.25, 95% CI 0.10 to 0.58, *P* = 0.001
Revascularization status	13 studies, 1600 patients, pooled OR 0.37, 95% CI 0.24 to 0.57
**Serum biomarkers**	
S100B (μg/l)	2 studies, 113 patients, pooled SMD 1.06, 95% CI 0.64 to 1.48
NLRP3 (ng/ml)	1 study, 200 patients, median value 1.85 vs. 1.11, *P* = 0.03
NT-proBNP (pg/ml)^10^	1 study, 1039 patients, adjusted OR 1.421, 95% CI 1.164 to 1.735, *P* = 0.001
**Reperfusion interventions for prevention**	
IST-3 Collaborative Group	1 study, 2961 patients: intravenous alteplase was associated with an increased risk of malignant brain edema, but it was also associated with accelerated clearance of hypertense artery sign on brain CT (OR 0.67, 95% CI 0.50–0.91), which in return reduced brain swelling (OR 0.25, 95% CI 0.14–0.47).
MR CLEAN Investigators	1 study, 462 patients: successful reperfusion after endovascular treatment was associated with reduced middle shift (OR 0.25, 95% CI 0.12–0.53).
HERMES Collaborators	7 studies, 177 patients with large hemispheric infarction*: thrombectomy and reperfusion were both associated with functional improvement (common OR 2.30, 95% CI 1.32–4.00 for thrombectomy; common OR 4.73, 95% CI 1.66–13.52 for reperfusion) but not of the degree of midline shift (β −0.27; 95% CI −1.52 to 0.98; reperfusion β −0.78; 95% CI −3.07 to 1.50); in 76 patients with very large infarction**: thrombectomy was associated with greater midline shift (β 2.76; 95% CI 0.33–5.20) but not functional improvement (OR 1.71, 95% CI 0.24–12.08).

Note: ASPECTS: Alberta Stroke Program Early CT Score; BBB: blood–brain barrier; CI: confidence interval; CT: computed tomography; CTP: computerized tomographic perfusion; DWI: diffusion weighted imaging; HERMES: the Highly Effective Reperfusion evaluated in Multiple Endovascular Stroke Trials; IST-3: The third International Stroke Trial; MCA: middle cerebral artery; MD: mean difference; MR CLEAN: The Multicenter Randomized Clinical Trial of Endovascular Treatment for Acute Ischemic Stroke in the Netherlands; NIHSS: National Institute of Health Stroke Scale; NLRP3: nucleotide-binding oligomerization domain-like receptor family pyrin domain-containing 3; NT-proBNP: N-terminal probrain natriuretic peptide; OR: odds ratio; SMD: standardized mean difference.

*large hemispheric infarction: ischaemic core 80–300 ml on DWI or CTP, or ASPECTS ≤5 on non-contrast CT; **very large infarction: core volume >130 ml or ASPECTS ≤3.

The development of brain oedema after stroke is associated with the damage to the blood–brain barrier. Simard *et al*. found a sulfonylurea receptor 1 (SUR1)-regulated NC_Ca-ATP_ channel was involved in the development of brain oedema in rodent stroke models.^6^ Moreover, glibenclamide (an SUR1 inhibitor) has been shown to reduce the volume of infarction and swelling in rodent stroke models.^7^ There is a phase II clinical trial which showed intravenous glibenclamide reduced midline shift, and reduced death from brain oedema in patients with large hemispheric infarction (NCT01794182). A subsequent phase III trial is ongoing (NCT02864953). Neuroinflammation is another possible mechanism for brain oedema. Animal studies show that NOD-like receptor thermal protein domain associated protein 3 (NLRP3) is upregulated in ischaemic brain tissue, aggregates neuroinflammation, and is associated with neurovascular damage after stroke. A clinical study found that serum concentration of NLRP3, assessed within 24 hours after stroke (median time from onset to blood collection, 3 hours) was higher in patients who developed malignant brain oedema at a later stage than those without malignant brain oedema (1.85 vs. 1.11 ng/ml; *P* = 0.026); furthermore, NLRP3 level was positively correlated with neurological impairment (on the National Institutes of Stroke Scale [NIHSS]) score on admission (Spearman *ρ* = 0.18, *P* = 0.01), and the association between NLRP3 and brain oedema was attenuated (OR 1.47, 95% CI 0.88–2.46; *P* = 0.138) after adjustment for age and NIHSS score.^8^ These findings indicate a role for neuroinflammation and increased microvascular permeability in the development of malignant brain oedema. Identification of more novel biomarkers could improve the accuracy of prediction and provide new targets for treatment.

In summary, brain oedema is a common complication of acute ischaemic stroke and an important preventable cause of death. Reperfusion therapies and DHC benefit selected high-risk patients. Further studies are needed to clarify natural history and mechanisms, with an aim to inform greater precision in outcome prediction at an early stage, guide timely interventions for prevention and treatment, broaden the benefit of existing effective therapies, and provide novel treatment targets.

## Conflict of interest

None declared.
